# Supplementary material for: A de novo algorithm for allele reconstruction from Oxford nanopore amplicon reads, with application to CYP2D6
Source: Bioinformatics. 2026 Jul 20;42(8):btag535. doi: 10.1093/bioinformatics/btag535 (PMC13437021; doi:10.1093/bioinformatics/btag535)
Supplement: Supplementary file 1 [file btag535_supplementary_data.zip › Supplementary Materials and Figures.docx]

A *De Novo* Algorithm for Allele Reconstruction from Oxford Nanopore Amplicon Reads, with Application to *CYP2D6*

Scott D. Brown^1,*^, Lisa Dreolini^1^, Agata Minor^2,3^, Michelle Mozel^4^, Nancy Wong^2^, Sharon Mar^2^, Amanda Lieu^2^, Maimun Khan^2^, Amanda Carlson^2^, Monica Hrynchak^2,3^, Robert A. Holt^1,5,6,†,*^, Perseus I. Missirlis^4,7,†,*^

Supplementary Materials

**Supplementary Table S1 (Separate file).** Summary table of all inferred amplicons, length-clusters, and allele reconstruction attempts with read counts at each stage.

**Supplementary File S1 (Separate file).** All successfully constructed consensus sequences for all *CYP2D6* samples.

**Supplementary Figure S1. *CYP2D6* read coverage from short reads.** Screenshot from Geneious Prime. The length-cluster of short reads from NA17300 (40,717 reads with mean length 445 bases) was aligned to the *CYP2D6* reference sequence. Coverage is shown in blue, spanning the entire expected amplicon. Exons are marked with grey segments under the green *CYP2D6* gene. Coverage is variable, and the region of highest coverage (exon 4-6) yielded a single reconstructed consensus sequence.

**Alt-text**: Read pileup across *CYP2D6* from short reads, showing non-uniform coverage.

**Supplementary Figure S2. Alignment of long and short consensus sequences to *CYP2D6*.** Screenshots from Geneious Prime. For NA10005 (top) and NA17300 (bottom), short consensus sequences were obtained from the respective length-clusters of short reads. The sequences of these short consensus sequences match at least one of the long consensus sequences in each sample, with the exception of mismatches at the beginning and end of the sequences.

**Alt-text**: Alignment of consensus sequences derived from long and short reads, showing agreement in underlying sequence.

**Supplementary Figure S3. Dot plot of duplication-detecting consensus sequence and *CYP2D6*.** Screenshot from Geneious Prime. The consensus sequence from NA02016_ligate from the 3.5 kb duplication-detecting amplicon (y-axis) is compared to the *CYP2D6* reference sequence (x-axis), showing the consensus sequence supporting the region upstream and downstream of *CYP2D6* as would be expected in the event of an allele duplication.

**Alt-text**: Alignment dot plot showing sequence similarity of duplication-detecting consensus sequence and *CYP2D6* reference sequence, showing duplication covering the beginning and end of the reference.

 **Supplementary Figure S4. Suspected *CYP2D6-CYP2D7* hybrid allele sequence aligned to *CYP2D6* and *CYP2D7*.** Screenshots from Geneious Prime. The *CYP2D7*-locus-derived consensus sequence from NA17203_ligate is aligned to the *CYP2D6­* reference (top) and *CYP2D7* reference (bottom), with the sequence identity as the green histogram above the sequences. Regions of lower sequence identity to either reference are boxed in light blue. This consensus sequence appears to best support a *CYP2D6*-*CYP2D7* hybrid allele, with exon 1 from *CYP2D6*, and the remainder of the sequence from *CYP2D7*.

**Alt-text**: Hybrid allele consensus sequence aligned to *CYP2D6* and CYP2D7*,* showing agreement with exon 1 of *CYP2D6*, and exons 2-9 of *CYP2D7*.

**Supplementary Figure S5. Alignment of NA17203_ligate *CYP2D6*-*CYP2D7* hybrid allele sequence to **68* hybrid allele sequence.** Screenshots from Geneious Prime. (A) Our NA17203_ligate **68* allele sequence shows a perfect match to the **68* sequence identified from NA12878 identified by Rubben et al, (B) with the exception of a C deletion observed in our sequence within a homopolymer stretch. (C) When our **68* hybrid allele sequence is aligned to the *CYP2D6* reference sequence, this deletion falls between exons 3 and 4, and thus would not be expected to change the function of the resulting protein.

**Alt-text**: Comparison of **68* consensus and reference sequences, demonstrating congruity with published results.
